# Supplementary material for: The Evolution and Characterization of the RNA Interference Pathways in Lophotrochozoa
Source: Genome Biol Evol. 2024 May 7;16(5):evae098. doi: 10.1093/gbe/evae098 (PMC11114477; doi:10.1093/gbe/evae098)
Supplement: evae098_Supplementary_Data [file evae098_supplementary_data.zip › Supplementary_materials.zip!/selected_miRNAs/NoGe_selected_miRNAs/mirdeep_pdf_files/NMRB01000055.1_Notospermus_geniculatus_strain_Ushimado_scaffold55,_whole_genome_shotgun_sequence_2176.pdf]

Provisional ID	: NMRB01000055.1_Notospermus_geniculatus_strain_Ushimado_scaffold55,_whole_genome_shotgun_sequence_2176
Score total	: 2.2
Score for star read(s)	: -1.3
Score for read counts	: 0
Score for mfe	: 0.5
Score for randfold	: 0
Score for cons. seed	: 3
Total read count	: 1437
Mature read count	: 1257
Loop read count	: 0
Star read count	: 180

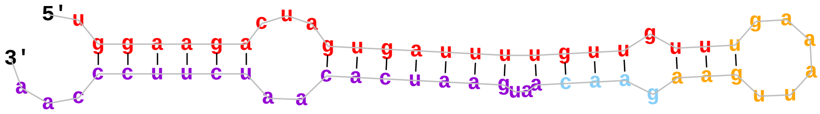

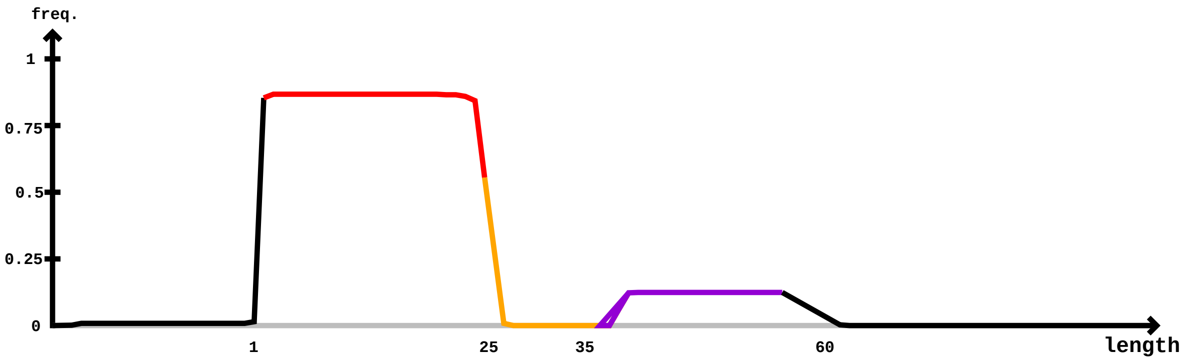

Mature Star

```
5'- cuuuucaggcagucgcagucuggaagacuagugauuuuguuguuugaaauugaagaacaaugaaucacaaucuucccaaauugugguugucacacccaacaaaccagauacac   -3'    obs
    cuuuucaggcagucgcagucuggaagacuagugauuuuguuguuugaaauugaagaacaaugaaucacaaucuucccaaauugugguugucacacccaacaaaccagauacac         exp
    .......((((((((((((..((((((...(((((((((((.(((......))).))))..)))))))..))))))...))))))))))))......................    reads    mm    sample
    .uuuucaggcagucgcaguc.............................................................................................    2    0    Mm3
    ....................uggaagacuagugauuuuguug.......................................................................    1    0    Mm3
    ....................uggaagacuagugauuuuguuA.......................................................................    1    1    Mm3
    ....................uggaagacuagugauuuuguugu......................................................................    12    0    Mm3
    ....................uggaagacuagugauuGuguugu......................................................................    1    1    Mm3
    ....................uggaagacuagugauuuuguuguu.....................................................................    16    0    Mm3
    .....................ggaagacuagugauuuuguuguu.....................................................................    1    0    Mm3
    .........................................................caaugaaucacaaucuuGccaa..................................    1    1    Mm3
    .........................................................caaugaaucacaaucuucccaa..................................    5    0    Mm3
    .........................................................caaugaaucacaaucuucccaU..................................    1    1    Mm3
    .........................................................caaugaaucacaaucuucccaGa.................................    1    1    Mm3
    .........................................................caaugaaucacaaucuucccaaa.................................    7    0    Mm3
    ..........................................................aaugaaucacaaucuucccaa..................................    9    0    Mm3
    ..........................................................aaugaaucacaaucuucccaaa.................................    2    0    Mm3

    cuuuucaggcagucgcaguc.............................................................................................    2    0    Fm3
    .uuuucaggcagucgcaguc.............................................................................................    4    0    Fm3
    ....................uggaagacuagugauuuuguugu......................................................................    10    0    Fm3
    ....................uggaagacuagugauuuuguuguu.....................................................................    13    0    Fm3
    .....................ggaagacuagugauuuuguug.......................................................................    1    0    Fm3
    .....................ggaagacuagugauuuuguuguu.....................................................................    1    0    Fm3
    .........................................................caaugaaucacaaucuCccca...................................    1    1    Fm3
    .........................................................caaugaaucacaaucuuccca...................................    7    0    Fm3
    .........................................................caaugaaucacaaucuuAccaa..................................    1    1    Fm3
    .........................................................caaugaaucacaaucuucccaa..................................    34    0    Fm3
    .........................................................caaugaaucacaaucuucccaaa.................................    5    0    Fm3
    ..........................................................aaugaaucacaaucuuccca...................................    7    0    Fm3
    ..........................................................aaugaaucacaaucuucccaa..................................    19    0    Fm3
    ..........................................................aaugaaucacaaucuucUcaa..................................    1    1    Fm3
    ..........................................................aaugaaucacaaucuucccaaa.................................    3    0    Fm3
    ...........................................................augaaucacaaucuucccaaau................................    2    0    Fm3

    ...................cuggaagacuagugauuuuguuguuu....................................................................    1    0    Mm2
```

```
                    Mature                                  Star
cuuuucaggcagucgcagucuggaagacuagugauuuuguuguuugaaauugaagaacaaugaaucacaaucuucccaaauugugguugucacacccaacaaaccagauacac
....................uggaagacuagugauuuuguug.......................................................................   2     0    Mm2
....................uggaagacuagugauuuuguugu......................................................................   29    0    Mm2
....................uggaGgacuagugauuuuguugu......................................................................   1     1    Mm2
....................uggaagacuagugauuuCguuguu.....................................................................   2     1    Mm2
....................uggaagacuagugauuuuguuguu.....................................................................   42    0    Mm2
....................uggaagacuagugauuuAguuguu.....................................................................   1     1    Mm2
....................uggaagacuagugauuuuguuguuuU...................................................................   1     1    Mm2
.....................ggaagacuagugauuuuguuguu.....................................................................   3     0    Mm2
.........................................................caaugaaucacaaucuucc.....................................   1     0    Mm2
.........................................................caaugaaucacaaucuuAccaa..................................   1     1    Mm2
.........................................................caaugaaucacaaucuucccaa..................................   11    0    Mm2
.........................................................caaugaaucacaaucuucccaaa.................................   11    0    Mm2
.........................................................caaugaaucacaaucuucccaaU.................................   1     1    Mm2
.........................................................caaugaaucacaaucuucccaaUu................................   2     1    Mm2
..........................................................aaugaaucacaaucuucccaa..................................   12    0    Mm2
..........................................................aaugaaucacaaucuucccaaa.................................   3     0    Mm2

....................uggaagacuagugauuuug..........................................................................   2     0    Mm1
....................uggaagacuagugauuuuguug.......................................................................   3     0    Mm1
....................uggaagacuagugauuuuguuU.......................................................................   1     1    Mm1
....................uggaagacuagugauuuuguugu......................................................................   12    0    Mm1
....................uggaagacuagugauuuuguuguu.....................................................................   84    0    Mm1
....................uggaagacuagugauuCuguuguu.....................................................................   1     1    Mm1
....................uggaagacuagugauuuCguuguu.....................................................................   1     1    Mm1
....................uggaagacuagugauuuuguuguuu....................................................................   2     0    Mm1
..........................................................aaugaaucacaaucuucccaa..................................   2     0    Mm1

.uuuucaggcagucgcaguc.............................................................................................   4     0    Fm1
...................cuggaagacuagugauuuuguu........................................................................   5     0    Fm1
...................cuggaagacuagugauuuuguugu......................................................................   3     0    Fm1
....................uggaagacuagugauuuug..........................................................................   1     0    Fm1
....................uggaagacuagugauuuuguu........................................................................   3     0    Fm1
....................uggaagacuagugauuCuguu........................................................................   1     1    Fm1
....................uggaagacuagugauuuuguug.......................................................................   10    0    Fm1
....................uggaagacuagugUuuuuguugu......................................................................   1     1    Fm1
....................uggaagacuagugauuAuguugu......................................................................   1     1    Fm1
....................uggaagacuagugauuuCguugu......................................................................   1     1    Fm1
....................uggaagacuagugauuuuguugu......................................................................   92    0    Fm1
....................uggaagacuagugauuGuguugu......................................................................   1     1    Fm1
....................uggaagacuaCugauuuuguuguu.....................................................................   1     1    Fm1
....................uggaagacuagugauuuuguuguu.....................................................................   168   0    Fm1
....................uggaagacuagugauuuAguuguu.....................................................................   2     1    Fm1
....................uggaagacuagugauuuCguuguu.....................................................................   1     1    Fm1
....................uggaagacuagugauuuGguuguu.....................................................................   1     1    Fm1
....................uggaagacuagugauuuuguuguuu....................................................................   4     0    Fm1
....................uggaagacuagugauuuuguuguuC....................................................................   1     1    Fm1
.....................ggaagacuagugauuuuguugu......................................................................   3     0    Fm1
.....................ggaagacuagugauuuuguuguu.....................................................................   8     0    Fm1
.....................ggaagacuagugauuuuguuguuu....................................................................   3     0    Fm1
..........................................................aaugaaucacGaucuucccaa..................................   1     1    Fm1
..........................................................aaugaaucacaaucuucccaa..................................   12    0    Fm1
..........................................................aaugaaucacaaucuucccaaa.................................   10    0    Fm1

....................uggaagacuagugauuuuguug.......................................................................   4     0    Fm2
....................uggaagacuagugauuuAguugu......................................................................   1     1    Fm2
....................uggaagacuagAgauuuuguugu......................................................................   2     1    Fm2
....................uggaagacuagugauuuuguugu......................................................................   243   0    Fm2
....................uggaagacuagugauuuGguugu......................................................................   3     1    Fm2
....................uggaagacuagugauuuCguugu......................................................................   1     1    Fm2
....................uggaagacuagugauuGuguugu......................................................................   1     1    Fm2
....................uggaagacuagugauuuAguuguu.....................................................................   4     1    Fm2
....................uggaagacuagugauuuuguuguG.....................................................................   1     1    Fm2
....................uggaagacuagugauuuuguuguu.....................................................................   392   0    Fm2
....................uggaagacuagugauuuGguuguu.....................................................................   2     1    Fm2
....................uggaagacuagugGuuuuguuguuu....................................................................   1     1    Fm2
....................uggaagacuagugauuuuguuguuu....................................................................   35    0    Fm2
....................uggaagacuagugauuuuguuguuuU...................................................................   11    1    Fm2
..........................................................aaugaaucacaaucuucccaaa.................................   7     0    Fm2
```
